# Supplementary material for: The genome sequence of the Marsh Grey, Eudonia pallida (Curtis, 1827) (Lepidoptera: Crambidae)
Source: Wellcome Open Res. 2026 Sep 28;11:775. [Version 1] doi: 10.12688/wellcomeopenres.27972.1 (PMC13662384; doi:10.12688/wellcomeopenres.27972.1)
Supplement: Peer review history

# Reviewer report: Reviewer response for version 1

**DOI:** 10.21956/wellcomeopenres.30774.r172484

**Published:** 2026-10-15

**Authors:** Ian M Carr

**Affiliations:** 1 University of Leeds, Leeds, UK

The report describes the sequencing of the Marsh Grey’s (Eudonia pallida) genome. The data was generated using the Tree of Life’s standard workflow and is described in their typical format. The reported genome appears to be high quality and worthy of indexation. The only issue is the writing style of the introduction which uses some unusual phrasing. While understandable, it could do with been edited by a second person to tighten it up.

Are the datasets clearly presented in a usable and accessible format, and the assembly and annotation available in an appropriate subject-specific repository?

Yes

Are sufficient details of the sequencing and extraction, software used, and materials provided to allow replication by others?

Yes

Are the rationale for sequencing the genome and the species significance clearly described?

Yes

Are the protocols appropriate and is the work technically sound?

Yes

Reviewer Expertise:

NGS data analysis, eDNA and genome assembly

I confirm that I have read this submission and believe that I have an appropriate level of expertise to confirm that it is of an acceptable scientific standard.

# Reviewer report: Reviewer response for version 1

**DOI:** 10.21956/wellcomeopenres.30774.r172489

**Published:** 2026-10-13

**Authors:** David EK Ferrier

**Affiliations:** 1 University of St Andrews, Scotland, UK

This genome report is part of the Darwin Tree of Life initiative under the umbrella of the Earth Biogenome Project. As such it uses rigorous and standardised techniques and protocols to enhance reproducibility across genome projects. All approaches are adequately described here, with suitable additional links and references to allow reproducibility and comparison across projects, in this case in the impressively sampled Lepidoptera.

Are the datasets clearly presented in a usable and accessible format, and the assembly and annotation available in an appropriate subject-specific repository?

Yes

Are sufficient details of the sequencing and extraction, software used, and materials provided to allow replication by others?

Yes

Are the rationale for sequencing the genome and the species significance clearly described?

Yes

Are the protocols appropriate and is the work technically sound?

Yes

Reviewer Expertise:

Genome

I confirm that I have read this submission and believe that I have an appropriate level of expertise to confirm that it is of an acceptable scientific standard.

# Reviewer report: Reviewer response for version 1

**Published:** 2026-09-28

**Authors:** Automated Benchmarking

**Affiliations:** 1 Wellcome Sanger Institute, Hinxton, UK

As this article is a Genome Note, Automated Benchmarking has been used to determine whether the reported assembly passes four defined checks established by the Earth BioGenome Project: the raw and assembled data are publicly available, the Earth BioGenome Project three-part metric is met, the k-mer completeness exceeds 90%, and the BUSCO score passes the stated threshold. For Eudonia pallida (ilEudPall1), all four checks passed. Data available: pass. ENA study PRJEB82177, raw-read accessions ERR13946466 and ERR13947486, and assembly accessions GCA_964340915.1 and GCA_964340885.1 are public. Earth BioGenome Project three-part metric: pass. Haplotype 1 is reported as 6.C.Q67, meeting the 6.C.Q40 reference standard. k-mer completeness: pass. The combined haplotype assemblies have 99.33% k-mer completeness, exceeding the Earth BioGenome Project target of 90%. BUSCO: pass. Haplotype 1, which is proposed as the reference assembly for this species, has 98.8% complete BUSCOs (98.4% single-copy and 0.3% duplicated). BUSCO software version: 5.5.0; lineage dataset: lepidoptera_odb10. It meets the target of greater than 90% complete and single-copy BUSCOs. This decision uses values reported in the Genome Note and public accession records.

Reviewer Expertise:

NA

I confirm that I have read this submission and believe that I have an appropriate level of expertise to confirm that it is of an acceptable scientific standard.
